# Comprehensive Analysis of the OASTL Gene Family in Potato (*Solanum tuberosum* L.) and Its Expression Under Abiotic Stress

**DOI:** 10.3390/ijms252313170

**Published:** 2024-12-07

**Authors:** Ting Tian, Jinyong Zhu, Zhitao Li, Weilu Wang, Minmin Bao, Xiaoqiang Qiu, Panfeng Yao, Zhenzhen Bi, Chao Sun, Yuanming Li, Zhen Liu, Yuhui Liu

**Affiliations:** 1 State Key Laboratory of Aridland Crop Science, Gansu Agricultural University, Lanzhou 730070, China; 18294510154@163.com (T.T.); zhujy_salvare@126.com (J.Z.); yaopf@gsau.edu.cn (P.Y.); 2 College of Agronomy, Gansu Agricultural University, Lanzhou 730070, China; lizt1225@163.com (Z.L.); wangwlcn@foxmait.com (W.W.); baomm_lucky@126.com (M.B.); qiuxxqq@126.com (X.Q.); bizz@gsau.edu.cn (Z.B.); sunc@gsau.edu.cn (C.S.); 3 College of Horticulture, Gansu Agricultural University, Lanzhou 730070, China; liyuanm@gsau.edu.cn

**Keywords:** cysteine synthesis, metal stress, OASTL gene family, potato, salt stress

## Abstract

O-acetylserine (thiol) lyase is a pivotal enzyme in plant cysteine biosynthesis, which is crucial for promoting plant growth, development, and resisting abiotic stress. However, the related studies on the potato OASTL gene family (StOASTL) have not been reported. In the present study, we identified 11 members of the StOASTL gene family, conducting a thorough analysis encompassing chromosome distribution, protein physicochemical properties, gene structure, protein-conserved motifs, and gene replication events. Phylogenetic scrutiny delineated these 11 StOASTLs into five distinct subfamilies. Using RNA-seq from the Potato Genome Sequencing Consortium (PGSC), we investigated the expression profile of *StOASTLs* in different tissues of DM (double-monoploid) potato and under abiotic/biotic stress, hormone treatment, and biostimulant treatment. The results showed that one of the StOASTLs (*Soltu09G024390*) was differentially expressed under different abiotic stresses and hormone treatments. Our findings showcased the differential response of one *StOASTL* (*Soltu09G024390*) to a spectrum of abiotic stresses and hormone treatments. *Soltu09G024390* was earmarked as a candidate gene and successfully cloned. Functional validation through yeast stress assays demonstrated that the heterologous expression of *Soltu09G024390* bolstered yeast tolerance to salt and cadmium stresses. This study provides a theoretical basis for revealing the role of the StOASTL family in potato response to abiotic stress and valuable insights for further study of the biological functions of StOASTL.

## 1. Introduction

Sulfur (S) is a vital nutrient element for plants, ranking closely behind nitrogen (N), phosphorus (P), and potassium (K) in importance, and it plays a crucial role in plant metabolism and development [1]. Inorganic sulfate is the most abundant sulfur-containing nutrient in plants, which is absorbed by plant roots and reduced and assimilated to cysteine in different organs of plant [2]. As the primary organic sulfur compound in plants, cysteine (Cys) is not only a precursor to the synthesis of glutathione (GSH), methionine, essential vitamins, thioesters, and other sulfur derivatives but also plays an irreplaceable role in various Redox signaling-related processes [3,4,5]. Cys is one of the critical amino acids in plants, essential to the functioning of any living cell [6]. Glutathione, a tripeptide composed of glutamic acid (Glu), cysteine (Cys), and glycine (Gly), is the primary endogenous antioxidant in plant and animal cells [7,8]. Moreover, cysteine is pivotal in alleviating the growth restrictions caused by abiotic stresses in various plants [9,10]. In plants, cysteine can be degraded to produce H_2_S, and low concentrations of H_2_S have been shown to participate in plant abiotic stress response [11,12,13,14,15]. Studies have shown that cysteine has the property of a reducing agent; L-cysteine can effectively mitigate the adverse effects of NaCl toxicity on the growth of flax plants by reducing lipid peroxidation, regulating the contents of total soluble sugar and proline, thereby improving the salt tolerance of flax plants [10]. It has also been found that cysteine and glutathione biosynthesis play a role in the detoxification of special epidermal cells, such as trichosomes, as a means of tolerance to different stresses [16].

O-acetyl serine (thiol) lyase (OASTL; EC 2.5.1.47) is a key enzyme in plant synthesis of cysteine, working with serine acetyltransferase (SAT; EC2.3.1.30) to regulate the cysteine biosynthesis [6,17,18]. SAT catalyzes the transfers of the acetyl-CoA to serine (ser), forming O-acetyl serine (OAS). At the same time, OASTL replaces the activated acetyl group of OAS with sulfide to produce cysteine [6,11,19,20]. OASTL is also essential in promoting plant growth and development and resisting abiotic stress [21]. OsOASTL-A1, as a cytoplasmic cysteine synthetase, catalyzes the conversion of OAS and sulfide into cysteine in rice and detoxifies cadmium (Cd) [22]. *Arabidopsis* (*Arabidopsis thaliana*) responds to salt stress by inducing transcription and translation of OASTL genes [2]. OASTL activity of the root of reeds and cattails increased under salt stress [23]. In *Arabidopsis* and tobacco, overexpression of OASTL led to moderate increases in cysteine (Cys) and glutathione (GSH) levels, resulting in enhanced tolerance to Cd [24,25,26,27,28]. It has also been shown that the increased rate of cysteine biosynthesis is responsible for the enhanced cadmium tolerance and accumulation in trichomes of leaves [29].

To date, the OASTL family has been identified in various plants, with 13 OASTL members in *Cardamine hupingshanensis* [21], 12 OASTL members in rice (*Oryza sativa*) [22], nine OASTL members in *Arabidopsis* [17,30], eight OASTL members in tomato (*Solanum lycopersicum*) [3], and seven OASTL members in sorghum (*Sorghum bicolor* L.), respectively [31], but there have been no reports on potato (*Solanum tuberosum* L.).

Potato is an annual herb of the genus *Solanaceae* [32] and native to the Andean regions of Peru and Bolivia [33]. Potatoes are widely planted worldwide due to their strong adaptability, high yield, and stability. Potato is the fourth largest food crop after rice, corn, and wheat. Abiotic stresses such as high temperature, drought, and saline-alkali seriously affect potato growth and are the main factors restricting potato yield [34]. OASTL is involved in many biological processes in plants, especially in plant resistance to abiotic stress. Therefore, it is significant to identify and analyze the characteristics and biological functions of the OASTL gene family at the whole genome level of potato.

In this study, a total of 11 members of the StOASTL gene family were identified for comprehensive analysis of biological information (chromosome distribution, protein physicochemical properties, gene structure, protein-conserved motif, and gene replication events). The expression of *StOASTL* genes in different tissues of DM (double-monoploid) potato and under abiotic/biotic stress, hormone treatment, and biostimulant treatment were analyzed using RNA-seq data from the PGSC. In addition, the yeast heterologous expression method analyzed the gene *Soltu09G024390*, which may be involved in salt stress response and metal stress response.

## 2. Results

### 2.1. Identification of OASTL Gene Family Members and Phylogenetic Analysis

In this study, BLASTP and HMM3.1 were utilized to screen for protein sequences containing the PALP domain (PF00291) across the potato whole genome (PGSC_DM_v6.1). The initially identified protein sequences were manually screened using SMART, Pfam, and NCBI databases, and sequences lacking a complete PALP domain were excluded. Subsequently, the candidate sequences were submitted to KEGG to screen o-acetylserine (thiol) lyase [EC 2.5.1.47]. This comprehensive approach identified 11 members of the StOASTL gene family. The length of the protein sequence, molecular weight, and theoretical isoelectric point of StOASTL gene family members were analyzed by the ExPasy. The results indicate that the protein lengths of the StOASTL gene family members ranged from 304 to 555 amino acids, with molecular weights varying from 32,194.33 to 59,388.74 kDa and theoretical isoelectric points ranging between 5.00 and 8.68 (Table S1).

**Figure 1:**
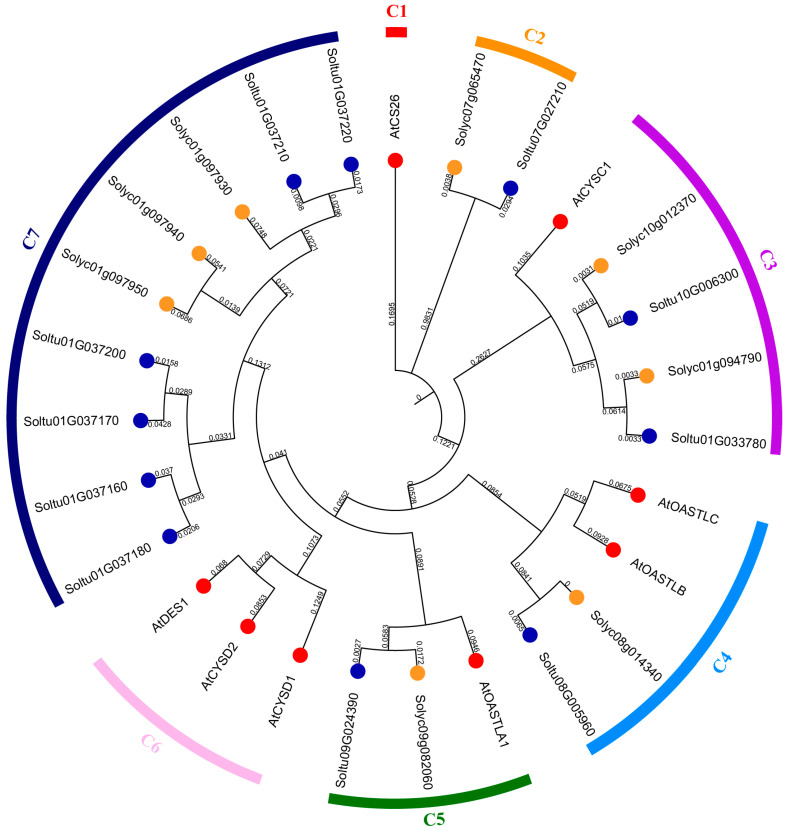
Phylogenetic tree analysis of OASTL gene family members of the *Arabidopsis*, tomato, and potato. Seven different subfamilies (C1, C2, C3, C4, C5, C6, and C7) are represented by red, orange, purple, light blue, green, dark blue, and pink, respectively. The red, orange, and blue circles represent OASTL members of the *Arabidopsis*, tomato, and potato, respectively.

To understand the evolutionary relationship among OASTL gene family members, a phylogenetic tree was constructed using the protein sequences of 27 OASTL family members from potato (StOASTLs), *Arabidopsis* (AtOASTLs), and tomato (SlOASTLs) (Figure 1). The results revealed that these 27 OASTLs could be divided into seven subfamilies (C1, C2, C3, C4, C5, C6 and C7). There is only one OASTL member (AtCS26) in the C1 subfamily. The C2 subfamily includes one StOASTL and one SlOASTL. The C3 subfamily comprises two StOASTLs, two SlOASTLs, and one AtOASTL. The C4 subfamily has one StOASTL, one SlOASTL, and two AtOASTLs. The C5 subfamily contains one StOASTL, one SlOASTL, and one AtOASTL. The C6 subfamily contains only three AtOASTLs. The C7 subfamily contains six StOASTLs and three SlOASTLs.

### 2.2. Chromosome Localization and Gene Replication Event Analysis of StOASTLs

**Figure 2:**
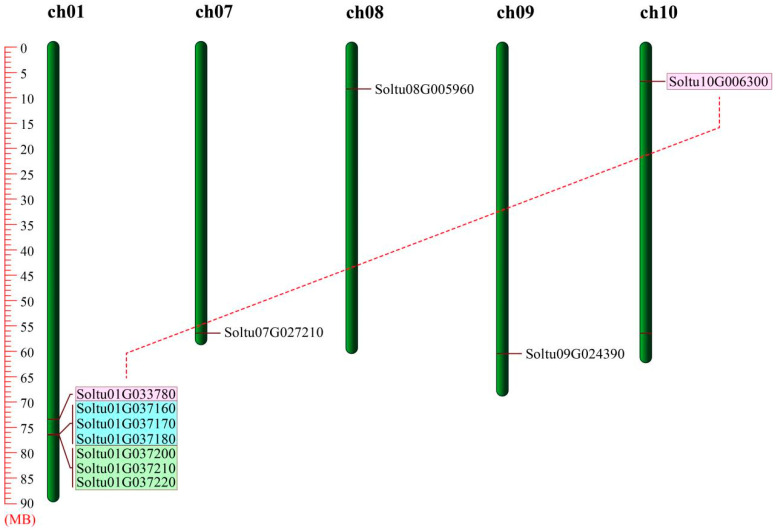
Distribution of *StOASTL* genes on chromosomes. The distribution of *StOASTL* genes on five chromosomes of potato. Tandemly duplicated genes are marked by blue and green boxes. The red line indicates the segmental duplicated genes.

The 11 *StOASTLs* were unequally distributed across 5 of the 12 chromosomes, as illustrated in Figure 2. Notably, all *StOASTLs* were positioned at the proximal or distal ends of chromosomes. The largest number of members were located in chromosome 1, including seven members, and chromosomes 7, 8, 9, and 10 each had one StOASTL member.

We analyzed gene replication events in *StOASTLs* using the MCsanX program. The results showed four tandem duplication pairs and one segmental duplication pair in the StOASTL gene family. Significantly, all tandem duplication pairs were located on chromosome 1, while the segmental duplication pair straddled chromosomes 1 (*Soltu01G033780*) and 10 (*Soltu10G006300*). These results suggested that tandem duplication events play a major role in expanding the StOASTL gene family, particularly within chromosome 1, which comprises 63.64% (7 out of 11 members) in the StOASTL gene family.

### 2.3. Analysis of Gene Structure and Conserved Motif of Potato OASTL Family

**Figure 3:**
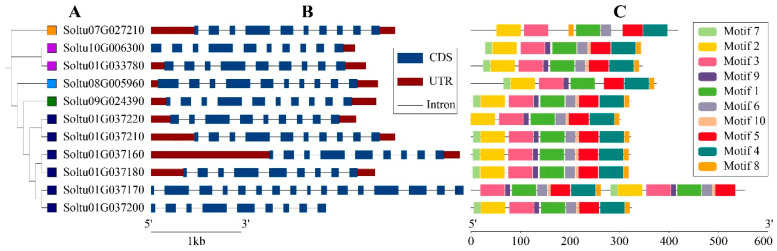
Evolutionary relationship, gene structure, and conserved motif distribution of St OASTL gene family members. (**A**) Evolutionary analysis of the potato OASTL family. The orange, purple, light blue, green, and dark blue squares represent the five subgroups C2, C3, C4, C5, and C7, respectively. (**B**) Exon/intron structure of StOASTL genes. The blue squares represent exons, the red squares represent the upper/downstream regions, and the black lines represent introns. (**C**) Conserved motif distribution of StOASTLs. The 10 different colored squares represent 10 different motifs.

The gene structure (exons and introns) of StOASTL family members was visually analyzed using GSDS 2.0 (Figure 3B and Table S1). The analysis revealed that among 11 *StOASTL* genes, five members (*Soltu07G027210*, *Soltu10G006300*, *Soltu08G005960*, *Soltu09G024390*, and *Soltu01G037210*) contained nine introns, five members (*Soltu01G033780*, *Soltu01G037220*, *Soltu01G037160*, *Soltu01G037180*, and *Soltu01G037200*) contained eight introns, and one member (*Soltu01G037170*) contained 15 introns.

We employed the MEME program (Version 5.5.5) to analyze the conserved sequences within StOASTLs, identifying ten motifs designated as Motif1~Motif 10. All of the 11 StOASTLs containedMotif1, Motif2, Motif3, Motif4, Motif5, and Motif8. Additionally, ten StOASTLs included Motif9 and Motf6, while nine StOASTLs contained Motif7 and Motif10. Apart from Soltu07G027210, Soltu08G005960, and Soltu01G037220, the remaining eight StOASTLs contained Motifs10. These results indicated that the protein sequences of the StOASTL family members were relatively conserved (Figure 3C and Table S2). Through multiple sequence alignment analysis, motif 2 and motif 3 were located in the N-terminal of PALP domain, and motif 1 and motif 6 were located in the middle of PALP domain. These four sites may be closely related to the function of the StOASTL family (Figure S1).

### 2.4. Analysis of Synteny Between Potato and Other Species

**Figure 4:**
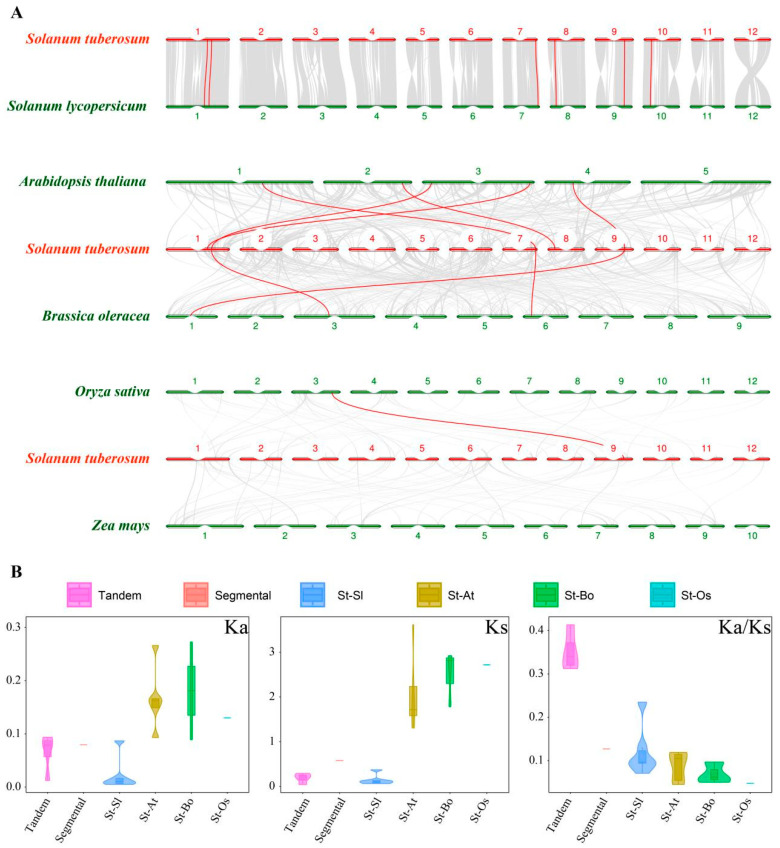
Homologous relationship of *StOASTL* genes among tomato, *Arabidopsis*, cabbage, rice, and maize. (**A**) The red lines indicate homologous *StOASTL* genes with multiple species (tomato, Arabidopsis, cabbage, rice, maize). (**B**) The Ka, Ks, and Ka/Ks values between potato and other species’ (tomato, *Arabidopsis*, cabbage, rice, maize) homologous genes. The horizontal coordinates represent tandem duplications, segmental duplications within potato, and homology between potato and tomato (St-Sl), *Arabidopsis* (St-At), cabbage (St-Bo), and rice (St-Os), respectively.

We analyzed the homology among OASTL genes in potato, tomato, *Arabidopsis*, cabbage (*Brassica oleracea*), rice, and maize (*Zea mays* L.) (Figure 4 and Table S3). The analysis revealed that the StOASTL genes have six, five, and three pairs of homologous genes with tomato, *Arabidopsis*, and cabbage, respectively. Since tomatoes and potatoes belong to the Nightshade family, they are closely related and have the most homologous genes. In contrast, there is only one homologous gene pair between potato and monocotyledonous rice and no homologous genes with maize.

Using KaKs Calculator 2.0, we calculated the Ka/Ks values for StOASTL segmental duplications, tandem duplications, and multispecies OASTL homologous pairs. The results showed that the Ka/Ks value for segmental duplications was 0.1361, and for tandem duplications, it ranged from 0.3122 to 0.4134. The Ka/Ks values for *StOASTL* and *AtOASTL* ranged from 0.0449 to 0.1190, for *StOASTL* and *SlOASTL* from 0.0707 to 0.2349, for *StOASTL* and *BoOASTL* from 0.0499 to 0.9667, and for *StOASTL* and *OsOASTL* it was 0.04779. All these Ka/Ks ratios were less than 1, indicating that these OASTL genes had evolved under purifying selection.

### 2.5. Analysis of StOASTLs Expression in Different Tissues of DM Potato

**Figure 5:**
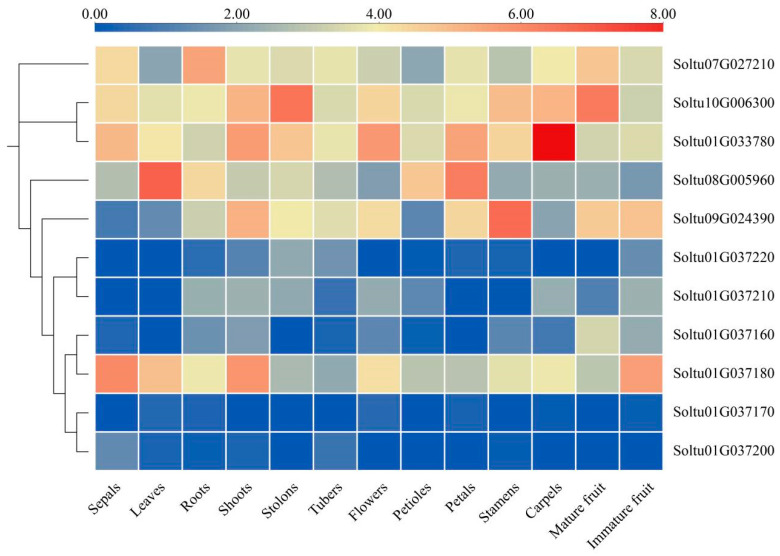
*StOASTL* gene expressions in different tissues of DM potato. In the heatmap, colors represent the log_2_FPKM of each gene.

To investigate the differential expression of potato OASTL gene family members in different tissues, we used RNA-seq data from the PGSC (Figure 5 and Table S4). The expression patterns of *StOASTL* genes in different tissues (sepals, leaves, roots, buds, stolons, tubers, flowers, petioles, petals, stamens, seedpods, mature, and immature fruits) were analyzed (Figure 5). The results showed that two *StOASTLs* (*Soltu01G033780* and *Soltu10G006300*) were highly expressed in all tissues (FPKM > 5). One *StOASTL* (*Soltu01G037170*) was low expressed in all tissues (FPKM < 1). In addition, some *StOASTLs* showed tissue-specific expressions. For instance, *Soltu01G33780* was specifically expressed in the carpel, *Soltu08G005960* in the leaf, and *Soltu09G024390* in the stamen.

### 2.6. Analysis of StOASTLs Expression Under Abiotic/Biotic Stress and Hormone Treatment

**Figure 6:**
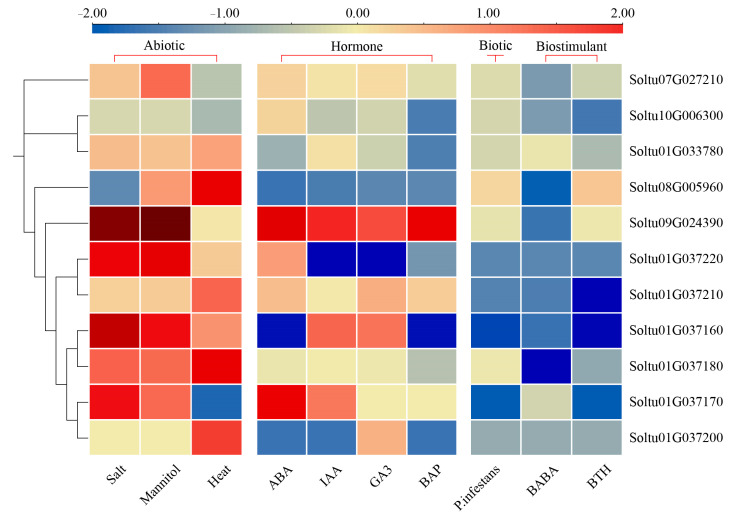
Expression of *StOASTL* genes under abiotic/biotic stress, hormone treatment, and biostimulant treatment. The color blocks represent the expression level using the log2FC value of each *StOASTL* gene.

Using RNA-seq data downloaded from PGSC, we analyzed the expression profiles of *StOASTLs* under three abiotic stresses (salt, mannitol, and high temperature), four hormone treatments (ABA, IAA, GA3, and BAP), two biostimulant treatments (BABA and BTH) and one biological stress (*P. infestans*) (Figure 6 and Table S5). The results showed that five, four, and three *StOASTLs* were differentially expressed under salt, mannitol, and high-temperature stress (|log_2_FC (fold change)| > 1 and FPKM > 1), respectively. Some of these genes respond to multiple abiotic stresses. For example, *Soltu09G024390* and *Soltu01G037220* were up-regulated under salt and drought stresses (log2FC > 1 and FPKM > 1), and *Soltu01G037180* was up-regulated in all three abiotic stresses. Six genes were differentially expressed under different hormone treatments. Three *StOASTLs* (*Soltu01G033780*, *Soltu10G006300*, and *Soltu01G037220*) were down-regulated only under BAP treatment (log2FC < −1 and FPKM > 1). One *StOASTL* (*Soltu01G037160*) was up-regulated under IAA and GA3 treatment. Two *StOASTLs* were differentially expressed under all hormone treatments, with *Soltu09G024390* up-regulated and *Soltu08G005960* down-regulated. Under the three biotic stresses, seven *StOASTLs* were differentially expressed. Among them, four *StOASTLs* (*Soltu07G027210*, *Soltu09G024390*, *Soltu08G005960*, and *Soltu01G037180*) responded only to BABA treatment. One *StOASTL* (*Soltu10G006300*) responded to BABA and BTH treatments. Two *StOASTLs* (*Soltu01G037160* and *Soltu01G037210*) responded to all three biotic stresses. It is worth noting that these StOASTLs were down-regulated under three abiotic stresses.

### 2.7. Cloning and Functional Analysis of StOASTL Gene

**Figure 7:**
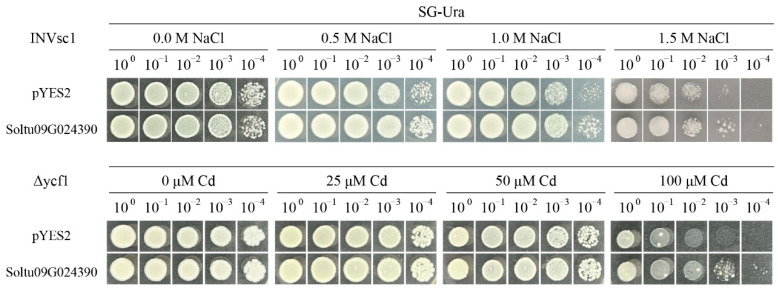
Growth phenotypes of transgenic yeast under salt and metal stresses. The growth phenotype of transgenic yeast overexpressing Soltu09G024390 was observed at 0 M, 0.5 M, 1 M, and 1.5 M NaCl concentrations and 0 μM, 25 μM, 50 μM, and 100 μM CdCl_2_ concentrations, respectively.

In order to confirm the potential involvement of the candidate *OASTL* gene (*Soltu09G024390)* in salt and metal stresses, we cloned the gene *Soltu09G024390* (978 bp) using the cDNA of potato variety “Qingshu-9” and inserted it into the yeast expression vector pYES2. The resulting construct, pYES2-*Soltu09G024390*, was introduced into the yeast strains INVSC1 and ∆ycf1, with the empty pYES2 vector (pYES2-EV) as the control and treated with different concentrations of NaCl and CdCl_2_ for 5 days (Figure 7).

Our findings indicated that, under normal conditions, the growth of transgenic yeast cells overexpressing pYES2-*Soltu09G024390* was comparable to that of the pYES2-EV cells. However, upon exposure to 0.5 M and 1.0 M NaCl treatments, the yeast cells overexpressing *Soltu09G024390* exhibited significantly improved growth compared with the pYES2-EV yeast cells. Even under the severe stress of 1.5 M NaCl, the yeast cells overexpressing *Soltu09G024390* maintained growth at dilutions of 10^−3^ and 10^−4^ times, while the pYES2-EV cells failed to do so. These results indicated that the overexpression of *Soltu09G024390* can enhance the salt stress tolerance of transgenic yeast cells. Furthermore, when subjected to CdCl_2_ treatment, the growth of yeast cells overexpressing the *Soltu09G024390* gene at concentrations of 25 μM and 50 μM CdCl_2_ did not show significant differences compared with the pYES2-EV cells. Furthermore, when subjected to CdCl_2_ treatment, the growth of yeast cells overexpressing the *Soltu09G024390* gene at concentrations of 25 μM and 50 μM CdCl_2_ did not show significant differences compared with the pYES2-EV cells.

## 3. Discussion

OASTL, a key enzyme in cysteine synthesis in plants, participates in various biological processes and plays an important role in plant growth, development, and resistance to abiotic stress [6,17,18,21]. Members of the OASTL gene family have been identified and analyzed in several species, such as *Arabidopsis* [17,30], rice [22], tomato [3], sorghum [31], and cardamom [21]. However, the study on the potato OASTL gene family has not been reported. In this study, 11 StOASTLs were identified at the whole genome level in potato. We conducted comprehensive analyses, including phylogenetic relationships, chromosome localization, gene duplication events, gene structure, conserved motifs, and response to abiotic stress.

The OASTL gene family is relatively conservative in plant evolution, with a similar number of family members and subfamilies across different species. In this study, 11 StOASTLs were divided into five subfamilies (C2-C4, C7) by phylogenetic analysis. This result is consistent with findings in other species. For example, 13 OASTL proteins in *Cardamine hupingshanensis* were grouped into four subfamilies [21], and seven OASTL proteins from *Sorghum bicolor* were also divided into four subfamilies [31]. Tandem and segmental duplication significantly promote the emergence of new family members and new functions in plant genome evolution [35]. The study found that StOASTL has four tandem duplication events (*Soltu01G037160* and *Soltu01G037170*, *Soltu01G037170* and *Soltu01G037180*, *Soltu01G037200* and *Soltu01G037210*, *Soltu01G037210* and *Soltu01G037220*) and one pair of segmental duplication genes (*Soltu01G033780* and *Soltu10G006300*). Notably, some tandem duplicate gene pairs were expressed inconsistently. For example, *Soltu01G037170* was down-regulated under heat stress but was not differentially expressed under BABA stimulation and low-expressed in 13 tissues (FPKM < 1), while Soltu01G037160 was not differentially expressed under heat stress. The expression of *Soltu01G037180* was down-regulated under BABA, BTH, and *P.infetans* treatment, and the expression of *Soltu01G037180* was high in 13 tissues. *Soltu01G037210* was not specifically expressed under salt and mannitol-induced drought stresses but was up-regulated under heat stress. It may be that gene duplication events that occurred during the evolution of the plant genome caused members of the StOASTL gene family to respond differently to stimuli.

Furthermore, an analysis of gene structure and conserved motifs of the StOASTL family suggested that the sequences of StOASTLs are relatively conserved. Homology analysis with multiple species revealed that some genes are very conserved in plant evolution and may play similar functions in different plants. For example, *Soltu09G024390* has homologous genes in tomato, *Arabidopsis*, cabbage and rice.

The OASTL proteins are critical in various plant biological processes [1]. By studying the expression patterns of genes in different tissues, the corresponding functions can usually be preliminarily predicted [36]. Previous studies have shown that AtCysC1 is involved in the formation of *Arabidopsis* root hair [37,38,39]. This study found that *Soltu01G033780* and *AtCysC1* were direct homologous genes; however, *Soltu01G033780* is not specifically expressed in the root, but in the carpella. This may be due to the differentiation of orthologous genes into different functions during plant evolution. Further research is needed to determine whether Soltu01G033780 is involved in the development of potato carpel. Furthermore, this study found that *Soltu09G024390* was specifically expressed in stamens, and *Soltu08G005960* was specifically expressed in leaves and petals. Whether these genes are involved in tissue development through cysteine biosynthesis remains to be further investigated.

The OASTL proteins play an important role in plant resistance to biotic/abiotic stress [31,40,41]. Studies have shown that O-acetylserine (thiol) lyase catalyzed the last step of cysteine biosynthesis, and OASTL overexpression can enhance plant tolerance to stress under stress conditions [3,26]. Previous studies have found that AtOASTL-A1 (At4G14880) is crucial for the synthesis of most cysteine in the roots and buds of *Arabidopsis*. It plays a major role in sulfur and cysteine metabolism and the plant’s antioxidant stress response [6,19,40,41,42,43,44,45]. Additionally, AtOASTL-A1 is involved in the response to abiotic stress [6,40,41,45]. Overexpression of *GmOASTL4* in tobacco can increase cysteine level and enhance tolerance to cadmium stress [46]. In this study, *Soltu09G024390* was homologous to *AtOASTL-A1*, which was up-regulated under salt and drought stresses. The above results indicated that Soltu09G024390 may have similar functions to AtOASTL-A1 under abiotic stress. In addition, *Soltu09G024390* was down-regulated under various hormone treatments (ABA, IAA, GA3, and BAP). Soltu09G024390 may be involved in the response of potato to abiotic stress through various hormone signal transduction pathways. Yeast is an effective system for assessing genetic tolerance [47]. Through yeast stress experiments, we found that overexpression of *Soltu09G024390* can enhance the tolerance of yeast to salt and cadmium stresses. It has been shown that the most effective strategy for plants to deal with the threat of toxic metals is to temporarily “deactivate” metal ions through GSH phytochelates (PCs) and metallothioneins (MTs), which is closely related to the H2S-Cysteine-centered sulfur metabolic pathway [48,49]. We hypothesized that Soltu09G024390 was involved in endogenous H2S production and cysteine synthesis under metal stress, thus improving plant tolerance to metal stress. This study also identified five members of the StOASTL gene family (Soltu01G037180, Soltu01G037220, Soltu01G037160, Soltu07G027210, and Soltu08G005960) involved in potato abiotic stress. These genes may be involved in the synthesis of cysteine and play an important role in the response of potatoes to abiotic stress. The specific mechanism needs to be further explored.

## 4. Materials and Methods

### 4.1. Identification and Physicochemical Analysis of the StOASTL Gene Family Members

The potato genome data (PGSC-DM-v6.1) were obtained from the Potato Genome Sequestration Consortium (PGSC, http://spuddb.uga.edu accessed on 5 June 2024) [32]. Genomic resources of *Arabidopsis* and tomato were acquired from Ensembl Plants (http://plants.ensembl.org/index.html accessed on 6 June 2024) [50]. Eight AtOASTLs protein sequences were retrieved from the TAIR (https://www.arabidopsis.org accessed on 7 June 2024) [51]. The HMM configuration file for the OASTL domain (PF00291) was acquired from the InterPro website [52]. Protein sequences containing the PALP domain (PF00291) were preliminarily screened across the entire potato genome using HMMER 3.1. All candidate sequences were submitted to SMART (http://smart.embl-heidelberg.de/ accessed on 11 June 2024) [53] and NCBI [32] for manual verification, and sequences lacking complete PALP domains were removed. Subsequently, the candidates were submitted to KEGG to identify o-acetylserine (thiol) lyases [EC 2.5.1.47] [54], allowing the identification of the potato OASTL gene family (StOASTLs). The amino acid length, molecular weight, and theoretical isoelectric point of StOASTL gene family members were analyzed using the ExPasy website [55]. Mapchart software [56] (version 2.32, http://www.wageningenur.nl/en/show/Mapchart.htm accessed on 13 June 2024) generated the chromosomal localization map of the StOASTL gene.

### 4.2. Construction of OASTLs Phylogenetic Tree

Phylogenetic trees were constructed using MEGA7.0 software. The analysis included eight AtOASTLs, eight SlOASTLs [3], and 11 StOASTLs, employing the maximum likelihood method (Poisson model, bootstrap was repeated 1000 times) [57]. Concurrently, a phylogenetic tree containing 11 StOASTL members was also constructed using the same method.

### 4.3. Analysis of Gene Duplication Events of StOASTLs and Homology with Other Species

MCScanX v1.5.1 was used for repeated identification and collinearity analysis of StOASTLs genes in potato [58]. Circos v0.69 [59] was used to visualize the homology relationships among OASTL genes in different species (including potato, tomato, *Arabidopsis*, cabbage, rice, and maize). Subsequently, the KaKs Calculator 2.0 [60] was used to determine the non-synonymous (Ka) and synonymous (Ks) substitution rates for each duplicate OASTL gene pair.

### 4.4. Characterization of Conserved Motif and Gene Structure of OASTLs

The conserved sequences of StOASTLs were subjected to further analysis using the MEME Suite (Version 5.5.5) [61] (https://meme-suite.org/meme/tools/meme accessed on 11 June 2024), enabling the identification of 10 motifs with lengths ranging from 6 to 50 amino acids. The gene structure of StOASTLs was visually depicted using the GSDS 2.0 tool [62] (http://gsds.gao-lab.org accessed on 12 June 2024).

### 4.5. Expression Analysis of StOASTLs

Using the published RNA sequencing data from PGSC Illumina RNA-seq assay (http://spuddb.uga.edu/ accessed on 13 June 2024) [32], the expression patterns of the OASTL genes in various tissues (sepals, leaves, roots, buds, stolons, tubers, flowers, petioles, petals, stamens, carpels, mature and immature fruits) of DM potato were comprehensively analyzed. Additionally, the expression profile of StOASTLs was examined under three abiotic stresses (salt stress: 150 mM NaCl; mannitol-induced drought stress: 260 μM mannitol; heat stress: 35 °C), four hormonal treatments [ABA (abscisic acid): 50 μM, 24 h; IAA (indole acetic acid): 10 μM, 24 h; GA3 (gibberellic acid): 50 μM, 24 h; BAP (benzylaminopurine): 10 μM, 24 h], two biostimulants treatment [BABA (β-aminobutyric acid): 24 h/48 h/72 h and BTH (Phenylpropyl thiadiazole): 24 h/48 h/72 h], and one biological stress (P. infestans: 24 h/48 h/72 h). TBtools v2.030 [63] software was used to draw the heat map.

### 4.6. Cloning of Candidate Genes

The coding sequences (CDSs) of the candidate gene Soltu09G024390 were amplified using “Qingshu NO. 9” cDNA as a template. The primers utilized in this amplification process are detailed in Table S6, and the resulting amplified sequences are depicted in Figure S2.

The gene amplification was performed in a 50 µL reaction mixture employing PrimeSTAR Max DNA Polymerase. Subsequently, the pYES2 vector was linearized with EcoRI and BamHI enzymes. The In-Fusion Snap Assembly cloning kit cloned the target gene into the vector.

### 4.7. Verification of Salt Tolerance and Heavy Metal Toxicity of Potato

The pYES2-*Soltu09G024390* and pYES2-EV were introduced into yeast strains INVsc1 and ∆ycf1 and cultured on SD Ura medium at 28 °C for 3 days. Single colonies were selected and confirmed as positive by PCR. For the stress experiment, transformed yeast cells were incubated in SG-Ura liquid medium at 28 °C for 16 h (OD ≈ 0.6). The cells were then serially diluted tenfold with sterile water (1, 10^−1^, 10^−2^, 10^−3^, 10^−4^) and spotted onto SG-Ura medium containing NaCl (0 M, 0.5 M, 1.0 M, 1.5 M) and CdCl_2_ (0 μM, 25 μM, 50 μM, 100 μM). The plates were incubated at 28 °C for 5 days and observed.

## 5. Conclusions

In this study, we comprehensively analyzed the phylogeny, gene structure, protein-conserved motifs, tissue-specific expression, and expression patterns of the StOASTL gene family under various biotic and abiotic stresses, as well as under multiple hormonal treatments. The results showed that StOASTL members were unevenly distributed on five chromosomes. Tandem duplication was the main driving force of StOASTL gene family expansion. In addition, by analyzing *StOASTL* gene expression in DM potato treated with abiotic stress and various hormones, we identified *Soltu09G024390* as a candidate gene involved in a plant’s abiotic stress response. The yeast stress experiment demonstrated that overexpression of *Soltu09G024390* could enhance the salt tolerance and resistance to metal stress in yeast cells. This study offers a theoretical framework for elucidating the involvement of the StOASTL gene family in potato responses to abiotic stress. It also provides valuable insights for the ongoing investigation of the biological functions of StOASTLs.

## Data Availability

Data are contained within the article and Supplementary Materials.

## Supplementary Materials

The following supporting information can be downloaded at: https://www.mdpi.com/article/10.3390/ijms252313170/s1.
