# Supplementary material for: Comprehensive Analysis of the OASTL Gene Family in Potato (Solanum tuberosum L.) and Its Expression Under Abiotic Stress
Source: Int J Mol Sci. 2024 Dec 7;25(23):13170. doi: 10.3390/ijms252313170 (PMC11641898; doi:10.3390/ijms252313170)
Supplement: Supplementary file 1 [file ijms-25-13170-s001.zip › Figure S2.pdf]

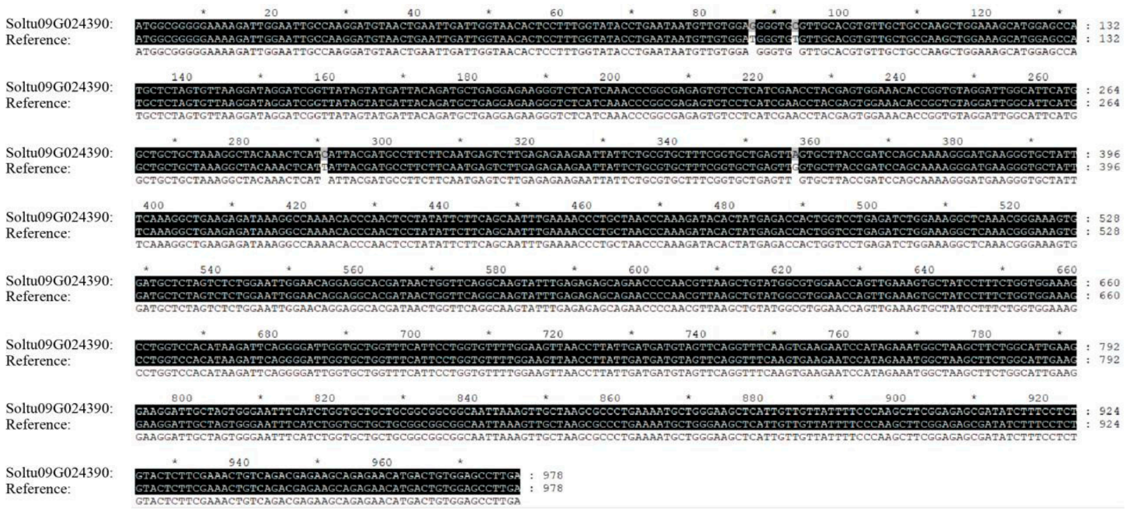

Figure S2. Sequence alignment of Soltu09G024390 gene.
