# Supplementary figures and images for: Comprehensive Analysis of the OASTL Gene Family in Potato (Solanum tuberosum L.) and Its Expression Under Abiotic Stress
Source: Int J Mol Sci. 2024 Dec 7;25(23):13170. doi: 10.3390/ijms252313170 (PMC11641898; doi:10.3390/ijms252313170)

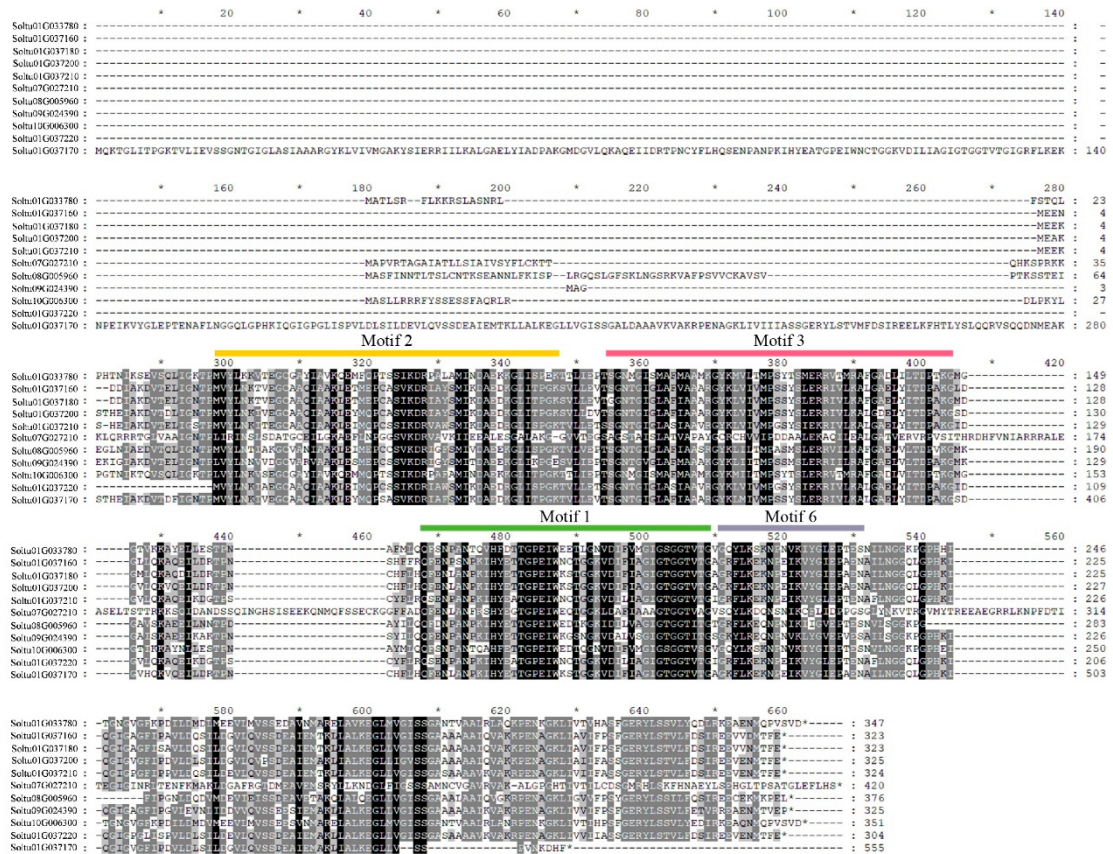

Figure S1. Multi-sequence alignment of StOASTL proteins.

Supplement: Supplementary file 1 [file ijms-25-13170-s001.zip › Figure S1.pdf]
